# Accuracy of different methods for blood glucose measurement in critically ill patients

**DOI:** 10.1590/S1516-31802009000500003

**Published:** 2010-02-03

**Authors:** Sérgio Antônio Pulzi, Murillo Santucci Cesar de Assunção, Bruno Franco Mazza, Haggéas da Silveira Fernandes, Mirian Jackiu, Flávio Geraldo Resende Freitas, Flávia Ribeiro Machado

**Affiliations:** I MD. Attending physician, Intensive Care Sector, Discipline of Anesthesiology, Pain and Intensive Care, Universidade Federal de São Paulo - Escola Paulista de Medicina (Unifesp-EPM), São Paulo, Brazil.; II MD. Medical coordinator, Intensive Care Sector, Discipline of Anesthesiology, Pain and Intensive Care, Universidade Federal de São Paulo - Escola Paulista de Medicina (Unifesp-EPM), São Paulo, Brazil.; III MD, PhD. Professor and Head of Intensive Care Sector, Discipline of Anesthesiology, Pain and Intensive Care, Universidade Federal de São Paulo - Escola Paulista de Medicina (Unifesp-EPM), São Paulo, Brazil.

**Keywords:** Hyperglycemia, Hypoglycemia, Sepsis, Norepinephrine, Shock, septic, Hiperglicemia, Hipoglicemia, Sepse, Norepinefrina, Choque séptico

## Abstract

**CONTEXT AND OBJECTIVE:**

Although glucometers have not been validated for intensive care units, they are regularly used. The aim of this study was to compare and assess the accuracy and clinical agreement of arterial glucose concentration obtained using colorimetry (Agluc-lab), capillary (Cgluc-strip) and arterial (Agluc-strip) glucose concentration obtained using glucometry and central venous glucose concentration obtained using colorimetry (Vgluc-lab).

**DESIGN AND SETTING:**

Cross-sectional study in a university hospital.

**METHOD:**

Forty patients with septic shock and stable individuals without infection were included. The correlations between measurements were assessed both in the full sample and in subgroups using noradrenalin and presenting signs of tissue hypoperfusion.

**RESULTS:**

Cgluc-strip showed the poorest correlation (r = 0.8289) and agreement (-9.87 ± 31.76). It exceeded the limits of acceptable variation of the Clinical and Laboratory Standards Institute in 23.7% of the cases, and was higher than Agluc-lab in 90% of the measurements. Agluc-strip showed the best correlation (r = 0.9406), with agreement of -6.75 ± 19.07 and significant variation in 7.9%. For Vgluc-lab, r = 0.8549, with agreement of -4.20 ± 28.37 and significant variation in 15.7%. Significant variation was more frequent in patients on noradrenalin (36.4% versus 6.3%; P = 0.03) but not in the subgroup with hypoperfusion. There was discordance regarding clinical management in 25%, 22% and 15% of the cases for Cgluc-strip, Vgluc-lab and Agluc-strip, respectively.

**CONCLUSION:**

Cgluc-strip should be avoided, particularly if noradrenalin is being used. This method usually overestimates the true glucose levels and gives rise to management errors.

**CLINICAL TRIAL REGISTRATION:**

ACTRN12608000513314 (registered as an observational, cross-sectional study)

## RESUMO

**CONTEXTO E OBJETIVO:**

Apesar de glicosímetros não serem validados para unidades de terapia intensiva (UTI), seu uso é corriqueiro. O objetivo foi avaliar a acurácia e concordância clínica entre a glicemia arterial por colorimetria (glicA-lab), glicemias capilar (glicC-fita) e arterial (glicA-fita) por glicosimetria, e venosa central por colorimetria (glicV-lab).

**TIPO DE ESTUDO E LOCAL:**

Estudo transversal realizado em hospital universitário.

**MÉTODO:**

Foram incluídos 40 pacientes com choque séptico e indivíduos estáveis, sem infecção. A correlação entre medidas foi avaliada tanto na amostra global quanto nos subgrupos em uso de noradrenalina e com sinais de hipoperfusão tecidual.

**RESULTADOS:**

A glicC-fita mostrou pior correlação (r = 0,8289) e concordância (-9,87 ± 31,76). Esta superou os limites aceitáveis de variação do Clinical and Laboratory Standards Institute em 23,7% dos casos, sendo maior que a glicA-lab em 90% das vezes. A glicA-fita teve a melhor correlação (r = 0,9406), com concordância de -6,75 ± 19,07 e variação significativa em 7,9%. Para a glicV-lab, obteve-se r = 0,8549, concordância de -4,20 ± 28,37 e variação significativa em 15,7%. Variação significativa foi mais frequente em pacientes com noradrenalina (36,4% *versus* 6,3%, P = 0,03), mas não nos com hipoperfusão. Houve discordância de conduta clínica em 25%, 22,5% e 15% dos casos para glicC-fita, glicV-lab e glicA-fita, respectivamente.

**CONCLUSÃO:**

O uso de glicC-fita deveria ser evitado, principalmente se há uso de noradrenalina. Geralmente, este método superestima a glicemia real e acarreta erros de conduta.

**REGISTRO DO ENSAIO CLÍNICO:**

ACTRN12608000513314 (registrado como estudo observacional transversal)

## INTRODUCTION

Severe sepsis and septic shock are the main causes of death in intensive care units. More than 750,000 cases of severe sepsis occur annually in the United States, amounting to 215,000 deaths/year in that country.^1^ Impaired microcirculation plays a leading role in this setting and, unless corrected, it can evolve to multiple organ dysfunction and death.^2^^,^^3^

Glucose homeostasis becomes modified in these patients, thereby resulting in insulin resistance, hyperinsulinemia and consequent hyperglycemia. This set of conditions is named stress diabetes, and it is a physiological response that ensures glucose supply to non-insulin-dependent tissues such as hepatocytes, nerve cells and alveolar, endothelial and immune system cells. Hyperglycemia is an independent predictor of adverse outcomes in cases of cardiovascular disease, neurological disorders, respiratory, liver and gastrointestinal disease, malignancy, sepsis and surgical patients.^4^^,^^5^ Normoglycemia is related to lower morbidity and mortality because of improvements in systemic inflammatory processes and in immune, endothelial and mitochondrial dysfunctions.^6^^,^^7^^,^^8^ Normoglycemic patients are less susceptible to bloodstream infection, renal failure, anemia and transfusion, polyneuropathy, hyperbilirubinemia and prolonged dependence on both mechanical ventilation and intensive care therapy.^9^^,^^10^ Additionally, glucose control is cost-effective.^11^

Thus, although glucose control is a priority in treating critically ill patients, glucose monitoring can be quite challenging. Considering that many intensive care patients are unable to express signs and symptoms of hypoglycemia, frequent and accurate measurements are pivotal.^12^ Given the low cost, easy sampling and prompt results of glucometers, capillary blood glucose levels are often determined using this method, although it has not been validated for intensive care patients.^5^ Critically ill patients have multiple relevant conditions that can interfere with measurements such as pH,^13^ partial pressure of oxygen,^14^ hematocrit,^15^ blood glucose levels^12^ and tissue hypoperfusion.^16^^,^^17^^,^^18^^,^^19^ Measurement mistakes may lead to unnecessary procedures regarding insulin doses and increase the risk of severe or prolonged hypoglycemia and its complications such as seizures, coma, arrhythmia and irreversible cerebral damage.^12^

## OBJECTIVE

This study aimed to compare capillary (Cgluc-strip) and arterial (Agluc-strip) blood glucose levels measured by a glucometer in critically ill patients, with their arterial blood glucose levels measured by means of colorimetry (Agluc-lab), which was considered to be the gold standard. Subgroups of individuals either using noradrenalin or presenting tissue hypoperfusion were also analyzed.

In addition, we also assessed the agreement with blood glucose levels measured through the central venous line by means of colorimetry (Vgluc-lab). Secondarily, we sought to determine whether the measurement method had any impact on the glucose level control procedures, based on a strict and well-established protocol.

## METHODS

### Type of study

This was a cross-sectional study carried out at a tertiary public institution. It was conducted in the intensive care unit of the Discipline of Anesthesiology, Pain and Intensive Care of Hospital São Paulo, Universidade Federal de São Paulo - Escola Paulista de Medicina (Unifesp-EPM). The research project had previously been analyzed and approved by the institution’s Ethics Committee. A free and informed consent statement was signed by all participating patients or their legal representatives.

### Sample

Forty patients were included based on the following inclusion criteria: age ≥ 18 years; presence of arterial and central venous lines; intensive glycemic control in accordance with the institution’s protocol and a signed informed consent statement. Patients with either diabetes or hemodynamic instability that was not solely related to sepsis were excluded.

Patients with two different profiles were recruited for the study. The first type consisted of patients in septic shock, on any dose of noradrenalin. The second type consisted of patients with no confirmed or presumed infection, who did not require noradrenalin for any reason whatsoever. Septic shock was defined in accordance with the 1992 consensus conference, as described elsewhere.^20^ In assessing tissue perfusion, hypoperfusion was defined as the presence of oxygen central venous saturation (ScvO_2_) lower than 70 mmHg and lactate higher than 20 mg/l.

### Procedures

A single set of tests was obtained per patient: Cgluc-strip, Agluc-strip, Vgluc-lab, Agluc-lab, arterial lactate and arterial and central venous blood gas determinations. Serum sodium, potassium, creatinine and bilirubin levels were obtained through the unit’s routine tests. Arterial and central venous lines were used for sample collection. The catheter lumen was washed with 10 ml of distilled water, followed by aspiration of 5 ml of blood to be discarded before the sample collection. The samples were immediately send to the laboratory and processed by a technician who was unaware of the sample origin. The Agluc-lab and Vgluc-lab measurements were each made using 3 ml of sample in tubes containing ethylenediaminetetraacetic acid (EDTA). This was done in the Olympus Au640e device, with hexokinase G-6-pyruvate dehydrogenase A (G-6-PDHA) reagent. Arterial and central venous blood gas and arterial lactate were determined by means of a microtechnique in the ABL 700 Series Radiometer® (Copenhagen, Denmark), on samples of 1 ml each in heparinized syringes. For Cgluc-strip, blood from the finger pad lanced with a 26G needle was used. Analysis was performed by the investigator immediately after sampling. The glucometer available at the institution was the Medisense FreeStyle Optium® (Abbott Laboratories, United States). The glucometer manufacturer’s manual specifies hematocrit as the only interfering variable, ranging between 15% and 65%.

The tests and clinical data were stored on a standardized case report form for each patient. Intensive glucose control was carried out in accordance with the institution’s protocol, which uses Cgluc-strip on a routine basis. The other blood glucose levels did not interfere with the intensive glucose control procedures that were used by the physician on call, except if Agluc-lab was lower than 40 mg/dl.

The Acute Physiology and Chronic Health Evaluation II (APACHE II) on admission to the unit and the Sequential Organ Failure Assessment (SOFA) on the sample collection day were calculated. There was no deadline for patient inclusion after the first organ dysfunction.

Analysis of results was performed on the full data set and also considering the subgroups defined above, i.e. patients with and without septic shock. Thus, patients in septic shock (on noradrenalin) were compared with those who were stable from a hemodynamic point of view (not using noradrenalin), regardless of the tissue perfusion, with the aim of assessing the possible effects of the vasoconstrictor on the agreement of the Cgluc-strip. Likewise, whether or not these patients were on noradrenalin, they were assessed in relation to the signs of tissue hypoperfusion (ScvO_2_ and lactate).

Assessment of the conformity of clinical management using the results provided by the different methods was based on the institution’s protocol, using the latest capillary glucose level for comparison. This analysis was performed in a blinded manner.

### Statistical methods

The sample size calculation was performed based on the correlation between Agluc-lab and Cgluc-strip, taking the null hypothesis to be absence of correlation, with r = 0.55 and, as an alternative hypothesis, the existence of a correlation with r = 0.80. Considering a significance level of 0.05 and power of 0.80 in a two-sided test, a sample of 38 patients would be needed.

Descriptive analysis of the population’s characteristics was carried out. The variables analyzed were subjected to the Shapiro-Wilk normality test. Results relating to continuous variables were expressed as means and standard deviations. Categorical variables were expressed as percentages and were subjected to the chi-square test. The results were considered statistically significant if P < 0.05.

Pearson’s coefficient was used to determine the correlation between the different measurements of glucose levels in relation to Agluc-lab, which was considered to be the gold standard.^21^^,^^22^ This correlation analysis between methods was based on standards established by the Clinical and Laboratory Standards Institute (CLSI). These standards state that two different methods for glucose level evaluation are equivalent if Pearson’s coefficient is greater than 0.9751.^21^^,^^22^

The agreement between the methods was established by means of the Bland and Altman test.^23^ The “bias” was defined as the mean difference between two measurements made by different methods; accuracy as the standard deviation of the “bias”; and the limit of agreement as two standard deviations (95%) of the “bias”. Furthermore, the CLSI defines that 95% of the results from non-laboratory measurements of glucose levels need to differ by less than ± 15 mg/dl in relation to the laboratory measurement, if the latter is < 75 mg/dl. If the laboratory measurement is ≥ 75 mg/dl, the variation needs to be less than ± 20% of the laboratory value.^21^^,^^22^

The Epi-Info and GraphPad Prism 5 statistical software were used.

## RESULTS

Forty non-consecutive patients were included in the study between October 2006 and April 2007. Of these, 26 (65.0%) were male. The reasons for the patients’ hospital admission were clinical disease in 12 cases (30.0%), emergency surgery in 15 (37.5%) and elective surgery in 13 (32.5%). The mean length of hospital stay at the time of inclusion was 3.7 ± 6.2 days. The mean age, APACHE II and SOFA were respectively: 55.3 ± 17.7; 15.5 ± 8.6; and 7.2 ± 4.3.

Noradrenalin was used for 24 patients and of these, 11 (45.0%) presented tissue perfusion abnormalities. Among the other 16 patients (without noradrenalin), only four (25.0%) presented tissue perfusion abnormalities (P = 0.158). Only one patient had mean arterial pressure (MAP) lower than 65 mmHg at the sample collection time. Among the 15 patients with tissue hypoperfusion, six had ScvO_2_ abnormalities (mean = 56.4 ± 18.4) and nine had lactate abnormalities (mean = 49.5 ± 22.8). None of the patients had concomitant ScvO_2_ and lactate alteration.

With regard to possible hematocrit interference with glucose level determinations by means of glucometry, the mean hematocrit level was 26.5% ± 4.3, with a minimum value of 17.0% and maximum of 36.0%, in agreement with the limits specified by the device manufacturer.

**Figure 1.**
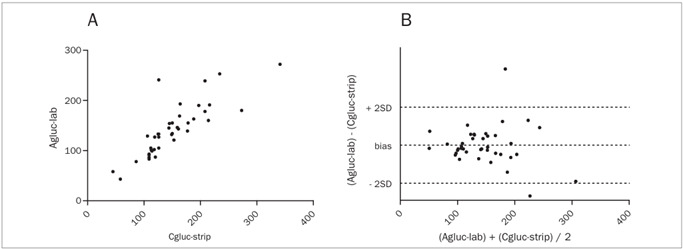
Pearson’s coefficient (1-A) and Bland and Altman’s test (1-B) between capillary blood glucose levels measured by glucometry (Cgluc-strip) and arterial blood glucose levels measured by colorimetry (Agluc-lab). Figure 1-A: r = 0.8289. Figure 1-B: bias = -9.87 ± 31.76 (-72.12 to +52.37). Full sample (n = 40). Blood glucose in mg/dl.

The mean glucose values were: Agluc-lab = 140.8 mg/dl ± 51.9; Cgluc-strip = 150.7 ± 55.8 mg/dl; Agluc-strip = 147.5 mg/dl ± 56.1; Vgluc-lab = 145.0 mg/dl ± 53.2. In Pearson’s correlation analysis, none of the blood glucose level determinations was satisfactory, in relation to Agluc-lab (Table 1). The worst correlation was with Cgluc-strip (r = 0.8289; P < 0.0001) (Figure 1-A). Bland and Altman’s method (Table 2) showed bias between Agluc-lab and Cgluc-strip equal to -9.8 mg/dl ± 31.7 (-72.12 to 52.37) (Figure 1-B). Cgluc-strip was higher than Agluc-lab in 26 patients (65.0%), with a mean variation of 25.7 mg/dl, while in the other 14 patients (35.0%), it was less than Agluc-lab, with a mean variation of 19.6 mg/dl. Analyzing the data according to the CLSI recommendations with regard to the acceptable percentage variation, only two Agluc-lab values lower than 75 mg/dl were found, which both had Cgluc-strip values within the acceptable limits of variation (± 15 mg/dl). However, when Agluc-lab was greater than or equal to 75 mg/dl (38 cases), Cgluc-strip was outside of the acceptable limits (± 20% of the Agluc-lab value) in nine cases (23.6%) (Table 3). In these nine cases, eight Cgluc-strip values overestimated the Agluc-lab. When Agluc-lab was higher than 150 mg/dl, such variation occurred in 28.5%.

**Figure 2.**
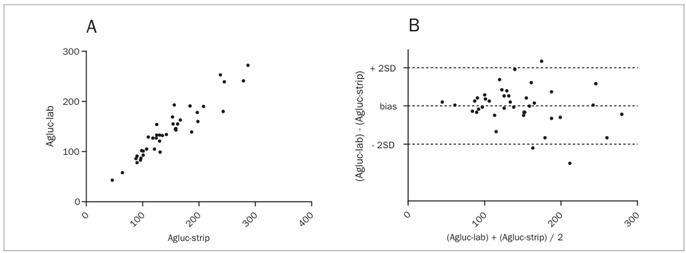
Pearson’s coefficient (2-A) and Bland and Altman’s test (2-B) between arterial blood glucose levels measured by glucometry (Agluc-strip) and arterial blood glucose levels measured by colorimetry (Agluc-lab). Figure 2-A: r = 0.9400. Figure 2-B: bias = -6.75 ± 19.07 (-44.13 to +30.63). Full sample (n = 40). Blood glucose in mg/dl.

**Figure 3.**
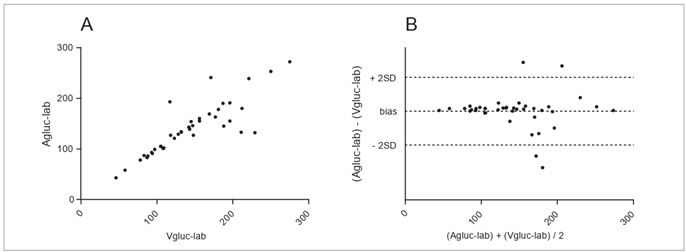
Pearson’s coefficient (3-A) and Bland and Altman’s test (3-B) between central venous blood glucose levels measured by colorimetry (Vgluc-lab) and arterial blood glucose levels measured by colorimetry (Agluc-lab). Figure 3-A: r = 0.8549. Figure 3-B: bias = -4.20 ± 28.37 (-59.81 to +51.41). Full sample (n = 40). Blood glucose in mg/dl.

Although unsatisfactory, Agluc-strip showed the best correlation with Agluc-lab (r = 0.9400; P < 0.0001) (Figure 2-A), with agreement in Bland and Altman’s test equal to -6.75 mg/dl ± 19.07 (-44.13 to 30.63) (Figure 2-B). Significant variation (CLSI criteria) was found in just three cases (7.5%), all with Agluc-strip higher than Agluc-lab. All patients had Agluc-lab greater than 75 mg/dl. In relation to Vgluc-lab, it presented r = 0.8549 (P < 0.0001) (Figure 3**-A**) and bias of -4.2 mg/dl ± 28.37 (-59.81 to 51.41) (Figure 3-B).

For Cgluc-strip, analysis of the noradrenalin subgroup showed that a significant percentage of measurements were outside of the acceptable limits of variation: 36.4% of the patients on noradrenalin and 6.3% of the patients not receiving noradrenalin (P = 0.03). On the other hand, there was no significant difference regarding tissue perfusion (35.7% and 16.7% in the subgroups with and without hypoperfusion, respectively; P = 0.17). With regard to the other methods, no significant difference was found in any of the subgroups. For these subgroups, the results from Pearson’s test and Bland and Altman’s test were similar to those for the full population, thereby showing no specific features (Tables 1 and 2).

Regarding clinical management, all of the methods led to some form of change in relation to Agluc-lab. The greatest change occurred with Cgluc-strip (n = 10.25%). For Agluc-strip, there was a management change for six patients (15.0%) and for Vgluc-lab, for nine patients (22.5%). These data and those for the subgroups are shown in Table 4.

**Table 1.** Correlation between arterial blood glucose levels measured by colorimetry and other methods

Method	Group				
	Full sample (n = 40)	Noradrenalin use		Tissue perfusion	
		Yes (n = 24)	No (n = 16)	Abnormal (n = 15)	Normal (n = 25)
Cgluc-strip	0.8289^*^	0.8246^*^	0.9612^*^	0.7983^†^	0.8951^*^
Agluc-strip	0.9400^*^	0.9526^*^	0.9175^*^	0.9541^*^	0.9454^*^
Vgluc-lab	0.8549^*^	0.9272^*^	0.6835^‡^	0.9253^*^	0.7918^*^

Cgluc-strip: capillary blood glucose levels measured by glucometry; Agluc-strip: arterial blood glucose levels measured by glucometry; Vgluc-lab: central venous blood glucose levels measured by colorimetry. Pearson’s correlation. ^*^P < 0.0001; ^†^P = 0.0004; ^‡^P = 0.035.

**Table 2.** Agreement between arterial blood glucose levels measured by colorimetry and other methods

Group	Method		
	Cgluc-strip	Agluc-strip	Vgluc-lab
Full sample (n = 40)	-9.87 ± 31.76 (-72.12-52.37)	-6.75 ± 19.07 (-44.13-30.63)	-4.20 ± 28.37 (-59.81-51.41)
Noradrenalin use			
*Yes (n = 24)*	-13.91 ± 37.91 (-88.23-60.40)	-11.20 ± 20.02 (-50.46-28.04)	4.08 ± 22.79 (-40.58-48.75)
*No (n = 16)*	-3.81 ± 18.78 (-40.63-33.01)	-0.06 ± 15.85 (-31.13-31.01)	-16.62 ± 31.97 (-79.30-46.05)
Tissue perfusion			
*Abnormal (n = 15)*	-7.24 ± 20.76 (-47.94-33.46)	-1.60 ± 15.36 (-31.71-28.51)	-6.08 ± 31.20 (-67.23-55.07)
*Normal (n = 25)*	-14.26 ± 45.14 (-102.74-74.21)	-15.33 ± 21.96 (-58.39-27.72)	-1.06 ± 23.60 (-47.32-45.19)

Cgluc-strip: capillary blood glucose levels measured by glucometry; Agluc-strip: blood glucose levels measured by glucometry; Vgluc-lab: central venous blood glucose levels measured by colorimetry. Bland and Altman’s test: bias ± standard deviation (limits of agreement). Blood glucose in mg/dl.

**Table 3.** Distribution of patients according to the Clinical and Laboratory Standard Institute (CLSI) recommendations regarding the acceptable percentage variation

Group	Method					
	Cgluc-strip		Agluc-strip		Vgluc-lab	
	≥ ± 20%^†^	< ± 20%^†^	≥ ± 20%^†^	< ± 20%^†^	≥ ± 20%^†^	< ± 20%^†^
Full sample (n = 38)^*^	9 (23.4)	29 (76.6)	3 (7.9)	35 (92.1)	6 (15.7)	32 (84.3)
Noradrenalin use						
*Yes (n = 22)*	8 (36.4)	14 (63.6)	2 (9.1)	20 (90.9)	2 (9.1)	20 (90.9)
*No (n = 16)*	1 (6.3)	15 (93.8)	1 (6.3)	15 (93.8)	4 (25)	12 (75)
P	0.03		0.62		0.18	
Tissue perfusion						
*Abnormal (n = 14)*	5 (35.7%)	9 (64.3)	2 (14.3)	12 (85.7)	2 (14.3)	12 (85.7)
*Normal (n = 24)*	4 (16.7%)	20 (83.3)	1 (95.8)	23 (4.2)	4 (16,7)	20 (83.3)
P	0.17		0.30		0.60	

^*^Excluding two patients with Agluc-lab < 75 mg/dl. Cgluc-strip: capillary blood glucose levels measured by glucometry; Agluc-strip: arterial blood glucose levels measured by glucometry; Vgluc-lab: central venous blood glucose levels measured by colorimetry. ^†^percentage of variation in relation to Agluc-lab. Chi-square test. Number of patients (%).

**Table 4.** Conformity in clinical management based on the institution’s protocol, using results provided by different methods in relation to the latest capillary glucose level

	Cgluc strip	Agluc-strip	Vgluc-lab
Full sample (n = 40)			
Management unchanged	30 (75.0)	34 (85.0)	31 (77.5)
Management changed	10 (25.0)	6 (15.0)	9 (22.5)
Noradrenalin use			
*Yes (n = 24)*			
Management unchanged	17 (71.0)	21 (87.5)	21 (87.5)
Management changed	7 (29.0)	3 (12.5)	3 (12.5)
*No (n = 16)*			
Management unchanged	13 (81.5)	13 (81.5)	10 (62.5)
Management changed	3 (18.5)	3 (18.5)	6 (37.5)
P	0.7110	0.6678	0.1198
Tissue perfusion			
*Altered (n = 15)*			
Management unchanged	18 (72.0)	21 (84.0)	18 (72.0)
Management changed	7 (28.0)	4 (16.0)	7 (28.0)
*Normal (n = 25)*			
Management unchanged	12 (80.0)	13 (86.0)	13 (86.0)
Management changed	3 (20.0)	2 (14.0)	2 (14.0)
P	0.7148	1.0000	0.4401

Cgluc-strip: capillary blood glucose levels measured by glucometry; Agluc-strip: arterial blood glucose levels measured by glucometry; Vgluc-lab: central venous blood glucose levels measured by colorimetry. Chi-square test. Number of patients (%).

## DISCUSSION

The results from this study suggest that Cgluc-strip should be avoided in an intensive care setting and that, when used, its results should be carefully interpreted. Its results could lead to improper management, thereby exposing the patients to a larger and prolonged number of hypoglycemic events.^16^^,^^17^^,^^18^^,^^24^^,^^25^

In studies conducted by van den Berghe et al., glucose control was carried out by means of Agluc-lab. In the first study^8^ on surgical patients, there were greater numbers of hypoglycemic events in the strict control group, in relation to the standard therapy group, from 0.8% to 5.1%. In the second study,^7^ this increase among clinical patients ranged from 3.1% to 18.7%. Thus, even with reliable glycemic measurements (Agluc-lab), patients undergoing intensive glucose control are more susceptible to hypoglycemic events and to related morbid events.^5^ The present study emphasizes that the use of Cgluc-strip may compromise accurate analysis on glucose values and increase the chances of hypoglycemic events. Moreover, in 20.0% of the cases (n = 8), Cgluc-strip overestimated the true glucose values. Cgluc-strip tended towards overestimating Agluc-lab, particularly in relation to extreme glucose values, and this had already been described in a previous study conducted by Kanji et al.^12^ These authors showed that, in hypoglycemic patients, variation beyond the acceptable limits occurred between Cgluc-strip and the reference method in 73.7%. Most of the time, Cgluc-strip overestimated the reference values, thereby delaying the diagnosis and hypoglycemia management. Such analysis was not performed in the present study, since there were only two cases of Agluc-lab < 70 mg/dl.

This study does not allow us to state whether the Cgluc-strip measurement error can be ascribed to the glucometer used, since only one type of device was used. Studies in the literature, using different devices, have shown significant Cgluc-strip errors and have suggested that such measurements should not be used in cases of critically ill patients.^12^^,^^16^^,^^17^^,^^18^^,^^25^ This downplays the idea that the error in glucose value measurement using a glucometer could possibly be related to the type of device used. Laboratory interference variables were eliminated. According to the literature, pH and pO_2_ values are not considered to be factors of relevance regarding interference.^13^^,^^14^ However, extreme hematocrit values can significantly interfere with Cgluc-strip measurements.^15^ Nevertheless, hematocrit values have been found to be within the limits permitted, for the device used, in all patients.^26^ Another factor giving rise to possible interference with Cgluc-strip would be the presence of edema in the extremities, which is a subjective clinical finding that was not analyzed at the time of sample collection in the present study. Kanji et al.^12^ assessed this matter and showed that the edema caused no error additional to what would be expected from this method.

The worst accuracy of Cgluc-strip, according to the CLSI criteria, was found in relation to patients on noradrenalin. In this subgroup, Cgluc-strip predominantly overestimated Agluc-lab, which places these patients at greater risk of hypoglycemia and more prolonged events, probably because they would receive a late diagnosis. This could have an impact on the evolution of and prognosis for critically ill patients, although so far there is no definitive evidence in the literature to suggest that accidental hypoglycemia would have an impact on mortality.^27^ Noradrenalin use has been found by several authors to have an influence on Cgluc-strip. In a retrospective study on 2,272 patients, Vriesendorp et al.^28^ assessed the risk factors for hypoglycemia using Agluc-lab and found that the use of a vasopressor was an important issue. They reached the conclusion that regardless of the glucose measurement method, patients with greater severity of illness were more subject to hypoglycemic events. In a multivariate analysis on situations that compromised the accuracy of Cgluc-strip, Critchell el al.^19^ showed that only the use of a vasopressor was significant. Kulkarni et al.^17^ analyzed 493 Cgluc-strip measurements, 75 of which from hemodynamically unstable patients, and showed that only in this group was there unacceptable accuracy. Another study, by Sylvain et al.,^18^ analyzed Cgluc-strip in 38 hemodynamically unstable patients, among whom 70% were on noradrenalin. The mean difference in relation to the reference method was 77 mg/dl. Atkin et al.^16^ also assessed unstable patients and found that Cgluc-strip presented acceptable agreement only in 36% of the measurements.

The present study also sought to correlate the accuracy of Cgluc-strip in relation to patients with signs of hypoperfusion, and this analysis differentiates this study from others in the literature. However, although the percentage of results above the acceptable limits was greater in the hypoperfusion group (35.7%), there was no significant difference in relation to the patients without any signs of hypoperfusion (16.7%). Such a difference would be expected, since there was a significant difference in relation to the group receiving noradrenalin, including similar percentages (36.4%). There may be many causes for such findings. The absence of statistical significance could perhaps be ascribed to the small size of the sample studied. Moreover, the methods chosen for hypoperfusion assessment (ScvO_2_ and lactate) were deficient, since they were less specific than the need for a vasopressor.

Agluc-strip was the method that was shown to be most representative of Agluc-lab. This is an important finding because most of the patients on noradrenaline had arterial lines available for sampling. As pointed out earlier, this was the group presenting the worst accuracy of Cgluc-strip, according to the CLSI criteria. Thus, in this group, arterial blood should be used for sampling whenever arterial lines are available, in order to minimize the risk of hypoglycemia.

Blood samples processed in a glucometer, except for capillary samples, have proven to be reliable in other studies.^12^^,^^16^^,^^18^ This suggests that the measurement error is predominantly related to the type of sample (capillary blood) rather than solely to the use of glucometers. Nonetheless, although Agluc-strip is, on a comparative basis, better than the laboratory method, its variation limits according to Bland and Altman’s test (-6.75 mg/dl ± 19.07) can be considered unacceptable from a medical point of view, particularly for patients at the lower limit of glucose levels (less than 70 mg/dl). Wide limits of variation were also found for Vgluc-lab (-4.20 mg/dl ± 28.37) and Cgluc-strip (-9.87 mg/dl ± 31.76), thus making its representativeness in relation to Agluc-lab questionable. This sounds odd, considering that both samples were processed using the laboratory method and expressed systemic characteristics. One possible explanation for this would be that some venous blood samples were contaminated by glucose solutions that were being administered through the sample collection route, although the collection technique used was appropriate for avoiding such occurrences.

This study has some limitations. First, patients were included on a nonconsecutive basis and, therefore, selection bias cannot be ruled out. Second, in the laboratory samples, glucose could have been used by red blood cells, thereby contributing towards the relative higher levels in the strip samples. However, this seems quite unlikely because the samples were immediately sent to the laboratory for processing. Third, the strip samples were not analyzed in a blinded fashion. However, because the analysis was performed by means of glucometry, the investigator was unable to interfere in the results.

## CONCLUSION

The results suggest that the use of Cgluc-strip in intensive care must be avoided, particularly if noradrenalin is being used. Predominantly, this method overestimates blood glucose levels, which implies procedural errors and exposes patients to more frequent and prolonged hypoglycemic events. Agluc-strip is the most representative method and it should be adopted as a technique for replacing the laboratory method whenever arterial lines are available. In all other patients, Cgluc-strip values should be routinely checked against laboratory values, particularly when the levels are at the lower limit of normality, in order to rule out hypoglycemia.
